# Game-Based Medical Education: Learning Effects of an Interdisciplinary and Interprofessional Escape Room

**DOI:** 10.1007/s40670-026-02661-3

**Published:** 2026-03-10

**Authors:** Lisa Stange, Torben P. P. Hornung, Tom Johann Schuster, Barbara Mueller, Elham Khatamzas, Renata Kraemer, Ali Zafar, Franziska Baessler

**Affiliations:** 1 Department of General Psychiatry, Center for Psychosocial Medicine, Heidelberg University Hospital, Thibautstrasse 4, Heidelberg, 69115 Germany; 2 Heidelberg Academy of Sciences and Humanities, Heidelberg, Germany; 3 Department of Infectious Diseases (Virology), Heidelberg University Hospital, Heidelberg, Germany; 4 Department of Infectious Diseases and Tropical Medicine, Heidelberg University Hospital, Heidelberg, Germany; 5 German Center for Infection Research (Deutsches Zentrum Für Infektionsforschung, DZIF), Partner Site Heidelberg University Hospital, Heidelberg, Germany; 6 Institute of Forensic Medicine and Traffic Medicine, Heidelberg University Hospital, Heidelberg, Germany; 7 Medical School Hamburg, University of Applied Sciences and Medical University, Hamburg, Germany; 8 Institute for Medical Education, Curriculum Development, and Educational Research at the Faculty of Medicine, Otto von Guericke University Magdeburg, Magdeburg, Germany

**Keywords:** Medical education, Medical didactics, Gamified learning, Game-based education, Escape room, Interdisciplinary learning

## Abstract

**Background:**

Innovative and interactive didactic methods in medical education have gained traction in recent years. This quasi-experimental study assessed the effectiveness of a Game-based interprofessional learning approach at a German medical school for knowledge gain and student satisfaction.

**Methods:**

An interdisciplinary escape room integrating learning goals from psychiatry, forensic medicine and infectious diseases was developed. Pre-post intervention questionnaires were used to determine differences in knowledge gain and evaluation between experimental and control groups. Knowledge retention was assessed one-month later using the same post-intervention questionnaire. Difference in pre- and post-test scores was determined as knowledge gain. Repeated Measures ANCOVA compared the variances of experimental and control group for pre- and post-test scores. Knowledge retention was analyzed correcting for pre-test scores, response delay and semester. Student satisfaction was measured with an evaluation survey consisting of 20 evaluation and three open-ended questions.

**Results:**

Sixty-two students (56.5% female, 43.5% male) participated in eight teaching interventions. ANCOVA showed a significant difference in knowledge retention between control and experimental groups (F(1,32) = 12.59, *p* = 0.001). No significant differences were observed in short-term knowledge gain (t(43.18)=-0.66, *p* = 0.511). Students evaluated the escape room as enjoyable and useful learning method to improve communication skills and team collaboration. Interprofessional aspects were perceived particularly positively.

**Conclusions:**

Escape room-based learning showed higher knowledge retention than control group. Escape room was perceived as more engaging and interprofessional than traditional teaching methods. Further research is needed with randomized controlled settings for deducing possible causal relationships.

**Supplementary Information:**

The online version contains supplementary material available at 10.1007/s40670-026-02661-3.

## Introduction

In medical education, the majority of teaching is still done via traditional passive learning methods such as lectures or seminars, which provide little room for active student participation. These methods are increasingly being supplemented with more interactive and engaging approaches such as bedside-teaching, working with simulated patients and problem-oriented learning [1–3]. An increasing number of studies on game-based learning (GBL) in medical education published since 2020 shows the growing interest in integrating more collaborative and engaging learning methods into the medical curriculum [4]. Studies suggest that GBL offers students a rich and immersive educational experience, supported by positive group dynamics [5, 6]. It has been shown to enhance academic performance by boosting motivation, increasing student engagement and fostering social interaction [5–7]. Studies also suggest that GBL can help students in improving analytic and clinical decision-making capacity, a crucial skill in transferring theoretical knowledge into clinical practice and integrating “knowledge, skills and critical thinking in patient care” [8, 9].

While there are various ways to teach using an escape room, the core idea is to design a scenario in which students complete a series of tasks either collaboratively or independently within a limited period of time [10]. Although educational escape rooms are traditionally done in person, they were effectively adapted to virtual environments during the COVID-19 pandemic, highlighting the versatility and adaptability of game-based teaching [4, 9, 11]. Studies indicate that participation in educational escape rooms leads not only to increased knowledge acquisition but also enhanced practical clinical skills, including physical examination techniques and essential non-technical and affective competencies such as active listening, teamwork, and reduced stigmatization towards specific patient groups [4]. Educational escape rooms have seen increasing global adoption [12] with successful implementations in medical education to train both students and professionals in multiple fields such as patient safety [13], nursing practice [14] and emergency medicine [11].

Theories of experiential learning, cognitive load and learner motivation allow the adaptation of escape room-based teaching in medical education. GBL sessions can be conceptualized as structured application of experiential learning, in which students progress through the four stages of Kolb’s experiential learning cycle: concrete experience, reflective observation, abstract conceptualization and active experimentation [15, 16]. The gameplay provides a time-pressured, clinically oriented concrete experience, a facilitated debriefing supports structured reflection while learning objectives promote conceptualization for future clinical encounters. The instructional design can also draw from cognitive load theory when clinical puzzles maintain an appropriate level of prior knowledge while minimizing minor distractions through clear guidelines and streamlined user interface [17].

In Germany, ongoing efforts aim to modernize the medical curriculum by shifting emphasis from subject-centered instruction towards competency-based learning in line with the National Competence-Based Catalogue of Learning Objectives for Undergraduate Medical Education (NKLM or Nationaler Kompetenzbasierter Lernzielkatalog Medizin) [18]. In medical schools, the curriculum is under revision for developing interdisciplinary and interactive courses to train students on patient-centered care, interprofessional education, and scientific competencies [19]. Although educators have emphasized upon modernizing medical education with innovative pedagogical strategies, the integration of GBL within curricula remains limited, with few documented implementations of gamified instructional methods. Moreover, existing studies have predominantly employed cross-sectional or exploratory designs, lacking the methodological rigor necessary to assess the long-term educational outcomes of these interventions [9].

Our earlier observational study based on a similar design and concept was well perceived and the findings showed immediate knowledge gains, calling for further research using an experimental design [20]. In this subsequent study, we designed and implemented an interdisciplinary medical escape room, integrating the national medical education learning goals from forensic medicine, psychiatry and infectious diseases (see Appendix A) [18, 19]. The escape room design was based on promoting experiential learning and self-determination, allowing teams a meaningful degree of autonomy in choosing strategies and distributing roles, to cultivate competence through progressively challenging but achievable tasks and structured feedback [21]. These design choices align with broader GBL and gamification literature, which indicates that well-aligned game elements can enhance motivation, persistence and learning outcomes in medical education. The gameplay incorporated multidisciplinary medical knowledge, practical competencies and responsibilities of a forensic physician, regarding identification of infectious diseases, its mode of transmission, psychiatric disorders and child endangerment. Using a quasi-experimental design, we compared learning outcomes of GBL with a traditional learning format for forensic medicine to observe group differences in learning and evaluation.

## Materials and Methods

This cross-sectional, quasi-experimental study assessed the learning outcomes of an interdisciplinary medical escape room and compared the differences in knowledge gain and student satisfaction between GBL group and a control group. The study was approved by the ethics committee of Heidelberg University Medical Faculty (S-840/2024) and conducted in accordance with the Declaration of Helsinki [22].

### Study Framework

The escape room was repeated eight times between March and July 2025. Using pre- and post-intervention questionnaires, we assessed the learning outcomes and feedback (evaluation) from medical students to determine perceived benefits of an innovative teaching approach over traditional teaching. One post-test questionnaire was completed by participants immediately after the interventions to assess short-term outcome (knowledge gain). Another post-test was implemented one-month later to assess long-term knowledge gain (knowledge retention). The questionnaires were administered online on the SoSci Survey platform and in paper format. Students who agreed to participate in the study completed the online pre- and post-tests under supervision before/after the intervention. The one-month follow-up test was completed online by students without supervision.

The experimental group participated in the escape room while the control group participated in a post-mortem teaching session, complemented with handout material on the escape room learning goals. The post-mortem inspection is an obligatory part of the medical curriculum, therefore all participants had to attend it. Participants of the experimental group could take part in the post-mortem inspection only after attending the escape room and completing the first post-test. To minimize group differences, students were recruited from the one-week clinical module of forensic medicine, whereby all students must take an obligatory exam in forensic medicine and therefore have studied the same material up to that point.

### Participants

Students enrolled in clinical semesters (semester 8 and above) and registered for compulsory forensic medicine module were invited to participate in the study. The activity was promoted using social media, official teaching platform (Moodle), onsite recruitment and via social contacts. Interested students could freely decide whether they wanted to participate in the control or intervention group. No randomization took place. Participation was voluntary and students were not graded for the activity.

Exclusion criteria were no enrollment in the forensic medicine module, lack of legal age (< 18 years) or lack of consent to the scientific use of data. Students had to confirm they participated only once in the escape room.

Since all students were registered for the compulsory forensic medicine module, they had the same access to lectures and took part in the mandatory postmortem teaching session.

### Pedagogical Settings

Before the intervention, the participants received an information leaflet and provided their informed consent, agreeing to the data privacy policy to start the pre-test questionnaire.

The escape room teaching session comprised 60-minute gameplay inside a simulated crime scene with students tasked with solving the mystery using medical knowledge puzzles, tasks and clues. The gameplay was followed by a 45-minute debriefing session with experts from different medical specialties and two police officers specialized in securing evidence at crime scenes. The debriefing session was used for giving feedback to students and allowing them to reflect on their learning experience [15, 23]. Students from the experimental group were only allowed to take part in the obligatory post-mortem inspection after having completed the escape room and the first post-test.

The control group only took part in a 60-minute postmortem inspection, which is a mandatory part of the regular medical curriculum. In addition, the control group received lecture slides covering psychiatric and infectiology topics that were covered in the escape room.

**Fig. 1:**
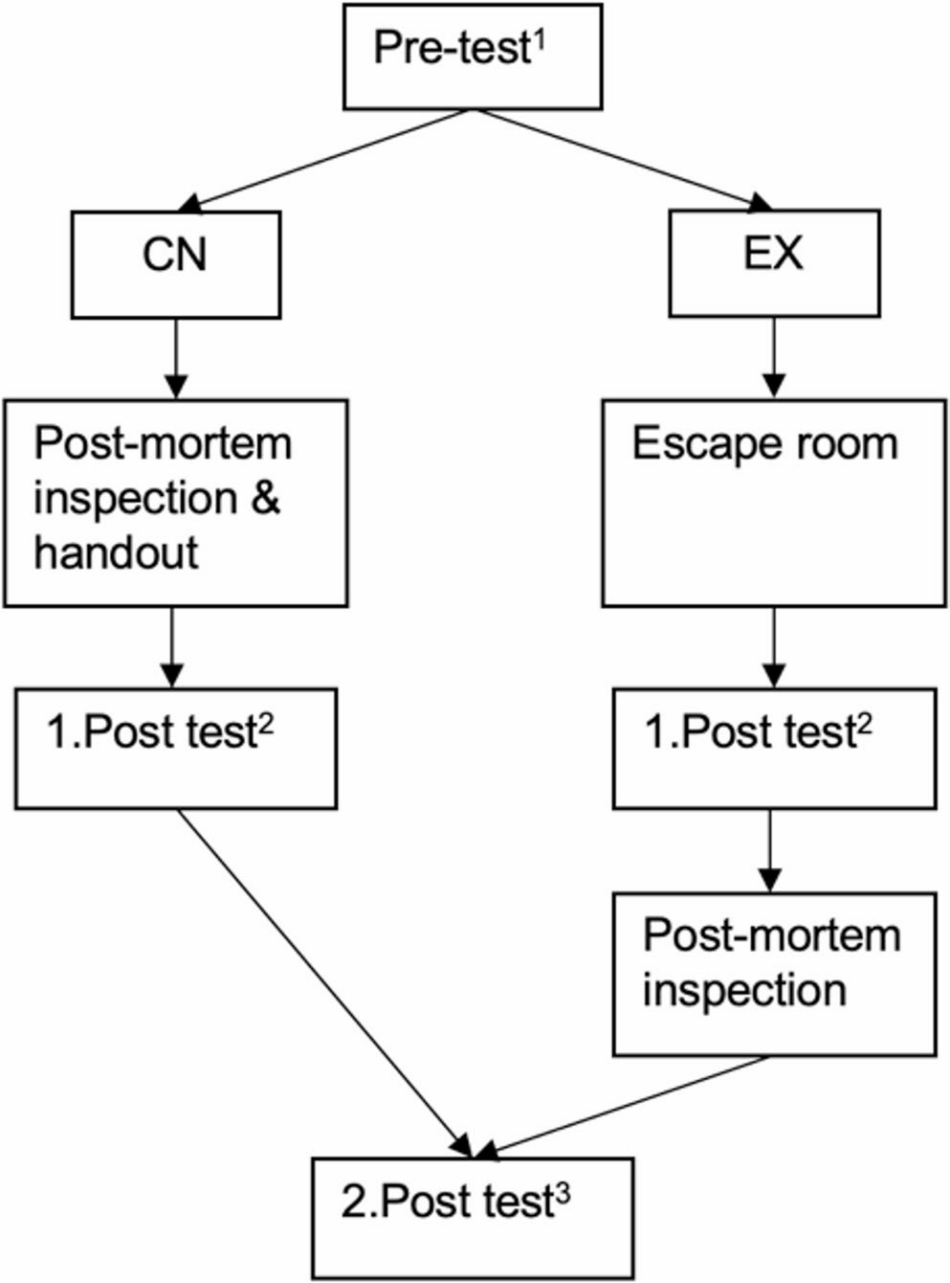
Study sequence. CN= Control Group, EX= Experimental Group. ^1^on-site MCQ, the test for the CN included 14 lecture slides at the end; ^2^on-site post-test right after learning module; ^3^ one month later, online

**Fig. 2:**
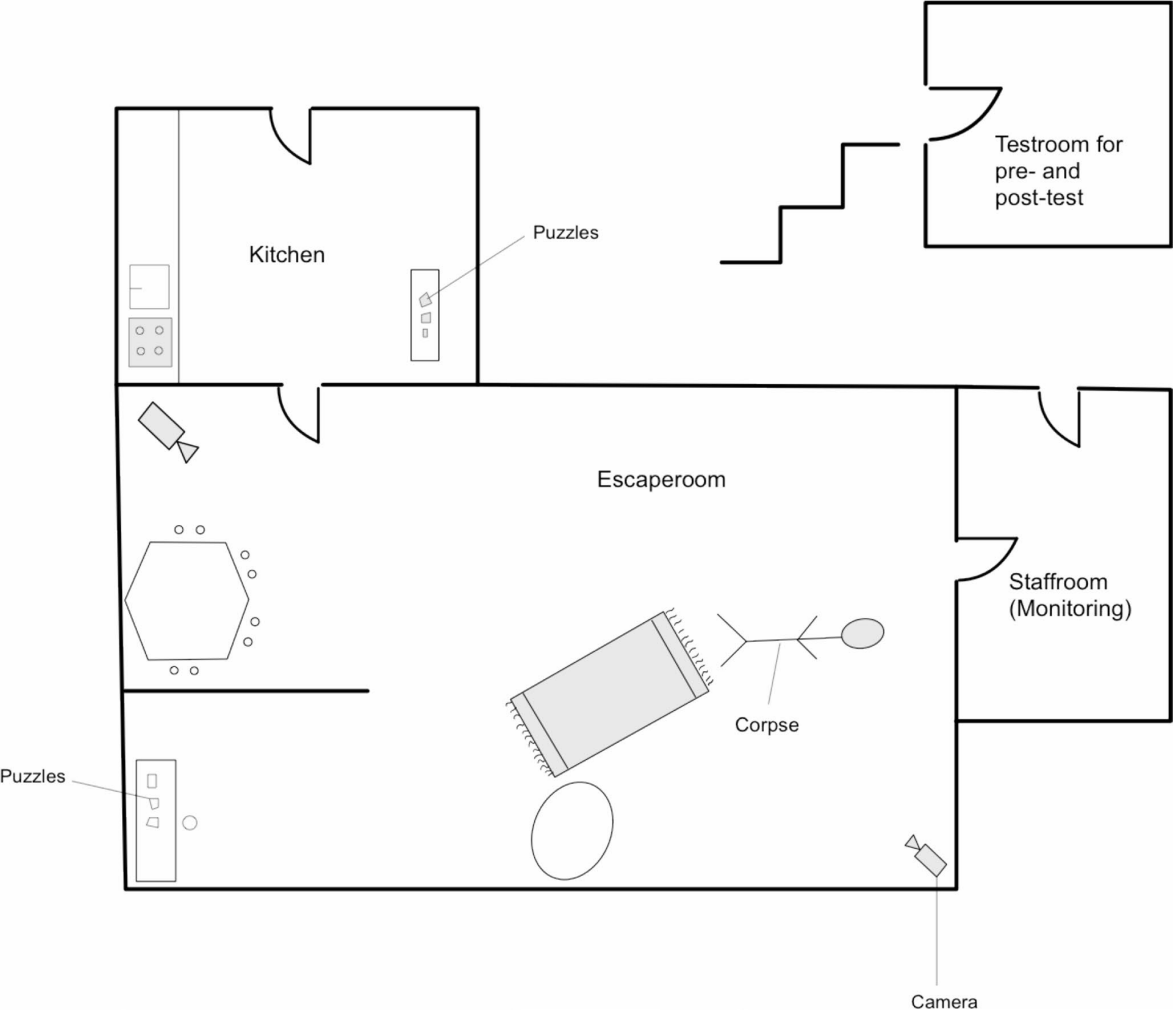
Sketch of the set-up of the escape room and surrounding facilities

Figures 1 and 2 illustrate the study sequence and setup of the escape room session, respectively. In Appendix A, we have described the escape room design, medical puzzles and learning goals. Further design details and the storyline are provided in Appendix B.

### Measurements

Pre-and post-test questionnaires consisted of the same 20 multiple-choice questions (MCQs) with five answer options each to measure objective knowledge before and after the interventions. Students scored one point for choosing each correct answer and one point for not choosing each incorrect answer, corresponding to a total score of 100 points.

Each question was derived from a specific learning goal described in the NKLM [18] (see Appendix A) and mainly referred to subjects taught in the clinical semesters. The MCQs were formulated after Delphi group discussions [24], involving two infectious diseases experts, one psychiatrist, two forensic doctors and two medical students. After two rounds of discussions, comments and adaptions, the MCQs were finalized with 100% consensus. The same knowledge-based questions were repeated in the two post-test questionnaires, one immediately after the intervention and one 30 days later.

**Fig. 3:**
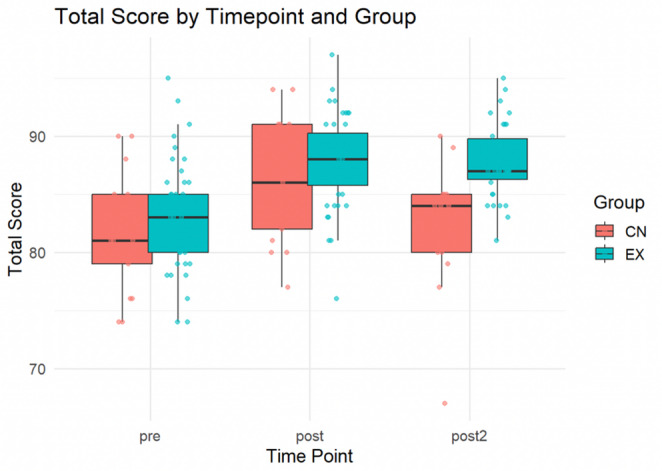
Boxplot depicting total test scores for the control group (CN) and experimental group (EX) over time

Another 20 questions were used to evaluate the two pedagogical interventions related to communication skills, perceived competency and teamwork skills. These evaluation questions were derived from instruments used in similar studies [25–27]. Each question was rated on a six-point Likert-like scale, ranging from 1 = strongly agree/very good to 6 = strongly disagree/very bad, corresponding to a score between 1 and 6 for each item.

To assess student satisfaction, three open-ended questions were used to obtain brief qualitative feedback on “suitable subjects”, “best aspects” and “improvements” for the escape room.

### Data Analysis

Data obtained on SoSci Survey and in paper format were combined for experimental and control groups and analyzed as one dataset in R. The paper-based questionnaires and the full code are available on request.

For pre-post knowledge assessment, the difference between scores of the pre- and first post-test was defined as “short-term knowledge gain”, expressed as means and standard deviations for each group. The “long-term knowledge gain” (retention) was assessed using the difference between the pre-test score and the one-month post-test score. We use the terms “*knowledge gain”* and “*knowledge retention”* as defined above throughout the paper.

Inferential statistics were used to measure differences in the experimental and control group scores related to gender, age and semester distribution. The knowledge gain for each group and the overall mean differences between the control and experimental groups were analyzed using Repeated Measures ANCOVA. To check the assumption of linearity between the covariates, they were plotted against the total score of the second follow-up post-test. Multicollinearity was tested by correlating all covariates. Normality was assessed using the Shapiro-Wilk-test and by visually inspecting the histogram of residuals.

As participants responded at different times to the one-month post-test, this resulted in an inter-individual time difference between the first and second post-test. This delay was defined as the number of days between the due date of the post-test (one-month) and the timestamp when the post-test was submitted. As this delay could influence the recall capacity [28], we included the pre-test scores and delay as covariates for correction. In addition to group assignment, pre-test scores as a baseline measure, semester and delay were included as covariates. Eta-square was used as a measure of effect size. The results were considered significant at *p* < 0.05.

Exploratory t-tests were performed to measure the in-group score differences between the different timepoints of the tests to better understand the dynamic of the test scores over time. A Bonferroni correction was done to adjust the significance level for exploratory t-tests.

For the 20 Likert-scaled evaluation questions, the responses between 1 and 6 were summed up for individual items to calculate means and standard deviations, offering an overview of satisfaction among participants and groups.

After an internal discussion, evaluation questions were also sent out to the control group. The evaluation survey, consisting of the same 20 evaluation questions and three open-ended questions, was sent out after all data had been collected. The control group was asked to evaluate the standard post-mortem inspection module to enable direct comparison between groups. The control group evaluation served as a baseline for the evaluation results of the experimental group. Mean evaluation scores of the experimental and control group were compared to identify possible benefits of the escape room approach over traditional teaching.

Responses to the three open-ended questions were qualitatively analyzed to obtain feedback on “suitable subjects for GBL”, “best aspects of escape room learning” and “improvements in game design”. Two authors (LS & FB) evaluated the responses from students and independently coded them to generate thematic categories via content analysis [29]. For intercoder agreement, the authors discussed their coding systems after the first round. The final categories were agreed upon through consensus building between the two coders. Only relevant categories and quotes were then translated to English to be included in this manuscript.

## Results

Overall, 62 medical students (56.5% female, 43.5% male; mean age = 23.6, min. 20, max. 32) enrolled between the 8th and 12th semesters participated in the study. The experimental group consisted of 45 students (72.6%) and the control group comprised 17 students (27.4%). As 24 students (EX = 17, CN = 7) did not respond to the one-month post-test, they were excluded from the knowledge retention analysis.

### Knowledge Assessment

The mean pre-test score of the experimental group was 82.7 out of 100 ((95% CI [81.3, 84.1]), SD = 4.56) and the mean post-test score was 87.8 ((95% CI [86.6, 89.0]), SD = 3.97). In the one-month post-test, the mean score was 87.8 ((95% CI [86.6, 89.1]), SD = 3.24). In the control group, the mean pre-test score was 81.6 ((95% CI [79.1, 84.2]), SD = 4.99) and the mean post-test score was 86.0 ((95% CI [83.3, 88.7]), SD = 5.21). The one-month post-test score was 80.4 ((95% CI [76.5, 84.3]), SD = 5.50).

ANCOVA showed a significant interaction between group (EX vs CN) and timepoint (pre-test vs. 1. post-test vs. 2. post-test), indicating differential changes in knowledge over time between groups (F(1,32) = 7.86, *p* = 0.009).

Table 1 summarizes all results.

A two-tailed Pearson correlation showed a negative association between pre-test scores and knowledge gain (*r* = − 0.49, t(42) = − 3.65, *p* = 0.001).

Group differences in covariates were assessed using t-tests, no differences were found (t_pre_(26.74) = − 0.77, *p* = 0.450; t_delay_(26.11) = 0.01, *p* = 0.995, t_semester_(30.71) = − 1.17, *p* = 0.250).

The Shapiro –Wilk test was significant (W = 0.90; *p* = 0.003), suggesting the residuals of data were not normally distributed. Visual inspection of the histogram of residuals indicated approximate normality apart from one extreme outlier. Therefore, the assumptions for ANCOVA were considered to be met.

A Welch two-sample t-test to assess group differences on knowledge gain (post-test minus pre-test) found no differences between the control and experimental group (t(43.18) = − 0.66, *p* = 0.511).

ANCOVA showed a significant main effect of group (F(1,32) = 12.59, *p* = 0.001) and pre-test score (F(1,32) = 7.00, *p* = 0.013) on knowledge retention when correcting for semester and follow-up delay. Neither semester (F(1,32) = 0.02, *p* = 0.893) nor follow-up delay (F(1,32) = 0.02, *p* = 0.883) showed a significant main effect. However, a significant delay × timepoint interaction was observed (F(1,32) = 9.94, *p* = 0.004), indicating time-dependent effects of delay. In addition, a significant interaction between group and timepoint was found (F(1,32) = 7.86, *p* = 0.009).Table 1 shows the results of ANCOVA. Figure 3 shows the boxplot of the test-scores for each group and timepoint.

**Table 1:** ANCOVA output with one-month post-test scores as dependent variable

Effect	MSE	F	*p*
Group	22.590	12.589	0.001**
Pre-test score	22.590	7.003	0.013*
Semester	22.590	0.018	0.893
Follow-up test delay	22.590	0.022	0.883
Timepoint (post, 2. post)	7.233	4.301	0.046*
Group × timepoint	7.233	7.863	0.009**
Pre-test score × timepoint	7.233	2.810	0.103
Semester × timepoint	7.233	0.505	0.483
Delay × timepoint	7.233	9.936	0.004**

* *p* < 0.05

### Evaluation

Escape room student group (*n* = 44) rated the overall experience with a mean score of 1.64 while the control group (*n* = 7) rated their overall experience with a mean score of 3.0. The experimental group rated all 20 evaluation questions higher than the control group, particularly related to overall enjoyment (1.18), interactivity (1.41) and suitability for long-term knowledge gain (1.64). Table 2 provides the mean scores for all evaluation items on a six-point Likert scale.

**Table 2:** Translated evaluation questions rated on a six-point likert scale (1 = very good/strongly agree, 2 = good/rather agree, 3 = satisfactory/agree, 4 = unsatisfactory/rather disagree, 5 = not good/disagree, 6 = very bad/strongly disagree); CN= control group (post-mortem inspection); EX= experimental group (escape room)

Question (six-point Likert scale)	Mean score EX (*n* = 44)	Mean score CN (*n* = 7)
1. Please rate the course overall.	1.64	3.0
2. Overall, I enjoyed the course.	1.18	3.40
3. The teaching format was suitable for enhancing my knowledge	1.86	2.80
4. The course increased my interest in the topics covered.	1.61	2.40
5. The session motivated me to further engage with the topics afterwards	1.93	3.20
6. The learning atmosphere during the teaching session was positive.	1.34	3.0
7. I consider the teaching format suitable for long-term knowledge retention.	1.64	4.0
8. The course helped me identify my weaknesses.	2.3	2.80
9. The teaching format promoted the active use of communication skills.	1.68	4.80
10. The teaching format supported teamwork (collaborative skills)	1.59	5.0
11. The teaching format encouraged me to apply leadership skills	2.23	5.0
12. The teaching format encouraged active participation	1.41	4.60
13. The topics covered are relevant for future physicians	1.32	1.80
14. This innovative session was challenging and stimulated reflection.	1.82	3.20
15. I would recommend this course to other students	1.30	3.40
16. The teaching format made me feel more competent in performing newly learned clinical tasks.	2.09	3.60
17. The teaching format helped reduce my anxiety about my future medical responsibilities	2.16	4.40
18. I sometimes feel overwhelmed by the thought of soon performing medical tasks independently.	2.27	2.20
19. After participating in this session, I feel better prepared for the medical profession	2.39	4.0
20. This innovative session promotes competencies in interprofessional collaboration	1.66	4.60

### Student Satisfaction

Open-text questions were answered by 28 participants (EX = 25, CN = 3). Five students mentioned emergency medicine as most suitable topic for GBL. Four students stated that all clinical subjects were suitable to be taught via escape room-based teaching. As best aspects of escape room learning, 10 students appreciated the interprofessional features, especially with non-medical experts such as police (*n* = 10) and the enjoyment factor of learning (*n* = 10). Five students rated positively the interactive nature of the escape room and teamwork. Some representative quotes on the best learning aspects included “the interdisciplinarity and the team work with fellow students”, “the playful approach to learning” and “the expertise of the police officers”.

For suggested improvements, 8 students stated that big group size (group size varied between 4 and 10) hindered their learning experience, suggesting a maximum of 5–6 students per group. A thorough introduction to the concept of the escape room, rules and the storyline was recommended (*n* = 4). Better time management and shorter duration of teaching were also pointed out (*n* = 5).

## Discussion

This quasi-experimental study compared the learning outcomes of an interprofessional escape room-based teaching activity with a conventional learning format for forensic medicine, building upon the results of our previous observational study in 2024 based on a similar design and concept [20]. Although there were no significant differences between the experimental and control groups in immediate knowledge gain, our results suggested significant improvement in knowledge retention over a period of one month. Moreover, students positively evaluated the escape room as a more suitable and enjoyable educational approach compared to the traditional postmortem teaching. The overall results suggest some effectiveness of escape room-based learning in enhancing long-term knowledge retention and underscore the value of interprofessional collaboration, which is essential in contemporary medical practice.

Our earlier research had shown promising results, indicating that escape room-based teaching had improved medical students’ knowledge while fostering a positive learning environment [20]. While the study resulted in significant immediate knowledge gains, its observational design and the absence of a control group limited our ability to draw comparative conclusions, calling for further research using an experimental design. Expanding on these findings, in this subsequent study we assessed not only the short-term knowledge gain, but also one-month knowledge retention associated with escape room-based learning. A control group was incorporated to enable direct comparisons between game-based and conventional teaching approaches. Based on the feedback from students in the earlier study, to make the escape room more practice-based, the gaming scenario and riddles were modified to include a criminal investigation and further learning goals from forensic medicine were added to the gameplay. We adopted an interdisciplinary and interprofessional approach by involving local law enforcement professionals as experts during the debriefing sessions. Therefore, the gameplay comprised multidisciplinary medical knowledge, practical competencies and responsibilities of a forensic physician. The pedagogical approach was based on promoting experiential learning and self-determination, allowing teams a meaningful degree of autonomy in choosing strategies and distributing roles, to cultivate competence through progressively challenging but achievable tasks and structured feedback [21]. These design choices align with broader GBL and gamification literature, which indicates that well-aligned game elements can enhance motivation, persistence and learning outcomes in medical education [17, 21, 32].

### Knowledge Assessment

Our latest findings demonstrated a moderate increase in knowledge gain following the escape room intervention as indicated by an immediate increase of 5.02 points from pre- to post-test in the experimental group. A comparable gain was observed in the control group with an immediate increase of 4.35 points between pre- and post-tests. Both groups exhibited improved post-test performance, with no statistically significant differences in short-term knowledge gain between them. Similar improvements in learning outcomes through game-based teaching have been documented in other medical disciplines [12–14, 30]. However, our findings might be explained by the higher pre-test scores of the experimental group (82.7 vs. 81.6), leaving less room for improvement. The negative correlation between higher pre-test scores and knowledge gain supports this assumption. The difference in the pre-test scores between the groups brings up the question of selection bias. Participants could freely choose in which group they wanted to participate, potentially leading to a bias with more motivated students choosing the escape room. This in turn could have led to the higher pre-test scores and the subsequently smaller improvement between the pre and post-test. The control group was composed of fewer participants than the experimental group, giving room to greater variance and less representative results.

The experimental group, however, had a significantly smaller decline in knowledge over time. After one month, knowledge retention scores decreased only slightly (-0.02) in the experimental group compared to a drop of 5.6 points in the control group. This might suggest that gamified learning leads to a more profound and retained learning that declines slower over time. Our control group was provided with similar content but only through a traditional learning approach, consisting of a practical postmortem examination supplemented by lecture slides representing the classical teaching approach. Earlier studies have also shown that gamified teaching can lead to deeper and more long-lasting learning effects [31]. Gamified approaches provide higher autonomy to students for choosing solutions for problems while group work can foster a sense of relatedness while learning, creating a more intrinsic motivated learning environment [32]. This is supported by the cognitive evaluation theory, which posits that intrinsic motivation is enhanced when the fundamental psychological needs for autonomy, competence and relatedness are fulfilled [21]. On the other hand, the cognitive load theory should be considered when designing medical escape rooms not to overload the limited capacity of working memory by minimizing task complexity and unnecessary cognitive demands [17].

If compared with a classical seminar or lecture without any interactive or practical aspects, we would expect a greater group difference in knowledge retention. One could argue that a standard lecture would have been more suitable as a control group to examine the benefit of an interactive and practical teaching method. However, the benefit of postmortem inspection as a control group was that the positive learning effect could be attributed to gamification rather than solely on interactive aspects. The standard postmortem inspection at this faculty is done with groups of students and one forensic doctor, demonstrating students and letting the students practice themselves. Thus, the control and experimental group resembled each other in group size, time for learning and practical teaching aspects.

### Evaluation

Overall, students evaluated the escape room positively, assigning it a rating of 1.64 out of 6, with 1 representing the highest mark. Even though the escape room was rated higher than the post-mortem inspection, these results must be interpreted with caution due to the small control group size (*n* = 17) and delayed distribution of the evaluation questionnaire. From an educational standpoint, students perceived the escape room as effective for long-term retention of learned content, promoting active engagement, addressing topics relevant to future medical practice and providing a stimulating challenge. These findings are consistent with prior studies suggesting that experiential learning in escape room settings enhances knowledge acquisition and recall capacity across various medical disciplines [26, 33, 34]. Game-based learning has been linked to improved educational outcomes, increased student motivation, stronger group dynamics and enhanced social interaction [5, 6]. Gamified teaching also holds the opportunity to practice crucial soft skills that are normally disregarded in traditional pedagogical approaches. While the medical profession asks for high levels of communication skills, team collaboration and leadership skills [35–37], often medical education does not fully meet these needs of future doctors who confront a variety of complex interpersonal situations with patients and colleagues requiring proficient communication skills, team collaboration and leadership [37, 38].

### Student Satisfaction

Despite few responses, most students emphasised the potentially broad use of gamified teaching with emergency medicine seen as a suitable topic of instruction. Students rated the escape room to be enjoyable and stimulating. Interactivity and teamwork as well as collaboration with police were perceived as particularly positive. In regular medical teaching, there is limited interaction with non-medical professions even though doctors work in a multi-professional environment. An understanding of these professions and their work is crucial to improve collaboration. For example, police officers are dependent on medicolegal officers to detect and document an unnatural cause of death to be informed and involved in further investigations. Therefore, an interprofessional approach to medical education may improve collaborations between different professions.

### Limitations

Some limitations in our study design are notable, as the study was neither randomized nor blinded, giving room for selection, performance and detection biases. The distribution between the control and experimental groups was highly uneven, which makes conclusions difficult for group comparisons. The intervention was offered as an optional extracurricular activity, and participation relied on students’ intrinsic motivation. For participation in the study no reimbursement was given, which made the recruiting process, specifically for the less engaging control group, difficult. Random assignment was not used, as it would likely have reduced overall attendance and engagement, compromising both recruitment and the ecological validity of the study. Instead, participants self-selected either the intervention or control condition, reflecting a quasi-experimental design commonly employed in educational research.

The riddles of the escape room were tailored towards the pre-defined learning goals of the study, whereas the postmortem inspection was not aligned with all learning goals. This could have led to higher post-test scores in the experimental group. However, the knowledge retention should not be influenced by this effect since no further teaching took place between the first and second post-test. Yet the relative decline between these two timepoints differs considerably between the two groups. Also, students might differ in their prior exposure to teaching content since participants were recruited from different clinical semesters. However, the pre-test should correct for this difference in prior knowledge. Since the one-month follow-up post-tests were done online from home, students might have used external sources during the tests. This would apply equally to both groups. The average time taken for the two post-tests did not suggest any major differences. A large number of students from both groups (EX = 17, CN = 7) did not respond to the one-month post-test questionnaire. Reminder emails were sent out to those who missed the deadline, leading to some responses with delays. This high drop-out rate might be due to lack of motivation or reimbursement for completing the study because the engaging part of the escape room or postmortem inspection was already completed. The completion of this online self-administered post-test was solely dependent on the students’ time and motivation and medical students are known to have little time or capacities for extracurricular activities. However, as the feedback for the escape room was generally positive, we do not assume a dissatisfaction with the study for not responding to the follow-up post-test survey. In the evaluation survey for question 14 and 20 the word *innovative* was used, which might have led to lower scores in the control group, since student did not see it as applicable. The overall course rating of the two teaching formats, when not using the word innovative, was nevertheless significantly higher for the experimental group.

## Conclusions

In this study, we evaluated an interdisciplinary escape room-based pedagogical approach for knowledge gain and student satisfaction. Our findings showed higher knowledge retention among students who participated in game-based learning versus a traditional instructional method. Students rated the escape room higher in terms of fostering teamwork and communication competencies. These results suggest that the primary strength of gamified learning may be the capacity to foster deeper knowledge retention, alongside beneficial secondary outcomes such as collaborative and communicative skill development.

Despite their pedagogical benefits, escape rooms remain a relatively novel approach within German medical education. To the best of our knowledge, this was the first interprofessional escape room conducted in collaboration with police representatives in a medical setting. Interaction between different medical specialties and other professionals such as police can be useful in forging interprofessional cooperation. Given the constantly evolving nature of medical instruction, a huge potential exists for innovative learning formats and research designs. Future research may compare learning outcomes of virtual and physical escape rooms, given the higher costs and resources required for in-person implementation. Studies may also examine the influence of escape room-based learning on team dynamics, communication and leadership development, as well as its applicability to clinical settings.

## Supplementary Information

Below is the link to the electronic supplementary material.

Appendix A

Appendix B

## Data Availability

Data collected and analyzed for this study are available and can be requested from the corresponding author on reasonable request.
