# Supplementary material for: Game-Based Medical Education: Learning Effects of an Interdisciplinary and Interprofessional Escape Room
Source: Med Sci Educ. 2026 Mar 10;36(3):1593–603. doi: 10.1007/s40670-026-02661-3 (PMC13355985; doi:10.1007/s40670-026-02661-3)
Supplement: Supplementary file 1 — Appendix A [file 40670_2026_2661_MOESM1_ESM.docx]

**Appendix A**

**Script of the Gameplay**

| **Time (min.)** | **Scene** | **Description** | **Tasks/Learning Goals** |
| --- | --- | --- | --- |
| 0-5 | Briefing | - general information given to the students: students are allowed to use the internet or any books lying around. They can explore the room freely, however there is a certain chronology to the story which will be moderated by some actors in the room. Things they cannot use will be marked accordingly.  -Setting: they are substitute general practitioners in a general practice. They are called to the house of one of the patients of the general practice. |  |
| 5-10 | Prologue | - Students are called/picked-up by two police officers.  - They are informed, that the police were called by a worried neighbor that has heard a fight next door. When they entered the flat, they found an unresponsive person lying on the floor. They then right away came up to ask the doctors team for help. -The students follow the police to the flat |  |
| 10-15 | Anamnesis | -Students must identify certain death signs to declare person as dead (The police confuses the students with uncertain death signs such as pulselessness)  -Students should wear gloves when first inspecting the corpse | Learning goal 1: identify certain death signs and declare death of person  Learning goal 2: wear appropriate clothing for post-mortem inspection (gloves)  NKLM learning goal: VIII.7.-02.6.4; VII.4.-01.2.1 |
| 15-25 | Post-Mortem inspection | -The police ask the students after they declared the death of the person to do a post-mortem inspection, students have a camera to take photos of their findings  -the police hand the students the death certificate to fill out | Learning goal 3: Do a proper postmortem inspection, which includes undressing the person completely  Learning goal 4: Properly filling out a death certificate  Learning goal 5: differentiating between the different causes and types of death and their legal consequences  NKLM learning goal: VIII.7.-02.6.4 |
| 25-30 | Organ donor and obduction | -the police find out that the person is an organ donor. They ask students if they should inform someone to get her ready as an organ donor.  -Later on, the police get called by the public prosecutor office, asking the students whether an obduction is implied and who’s responsibility it is to order an autopsy | Learning goal 6: to know the regulations for organ donors  Learning goal 7: understand who is responsible for ordering an autopsy and what is done in an forensic obduction.  NKLM learning goal: VII.2-02.3.7; VIII.6-04.4.11 |
| 30-40 | Injury-photo-word puzzle | -the police ask for the photo documentation of the student’s postmortem inspection to send them over to the forensic medicine department for some help. The Police officer comes back with photos and notes from the forensic medicine department, however on the way the order of the photos got mixed up. The students must sort out the photos and notes to be able to read the comments written by the colleagues. | Learning goal 8: identify different types of wounds. Differentiate between sharp and blunt force, self- and external inflicted injuries, identifying indicators for time and cause of death. NKLM learning goal: VIII.7.-02.6.4; |
| 40-45 | Infectiology and Vaccinations | -one of the police officers suddenly notices blood from the corpse on his hand and gets very worried about having caught any infectious disease. A bit anxious and worried, the officer asks the students if the person had any known infections, what the chances would be that he caught it and what he should do now.  -He then asks the students to check his vaccination pass since he isn´t sure if he has all necessary vaccinations. | Learning goal 9: knowing relevant infectious diseases and their transmission path. Learning goal 10: knowing  What to do in case of contact with potential infectious materials  Learning goal 11: correctly reading a vaccination pass and knowing the recommended vaccinations for people working with patients  NKLM learning goals: VII.1b-02.5.23; V.01.1.1.71 |
| 45-50 | Infectiology Crossword puzzle | Students find a crossword puzzle with infectious disease questions. The Keyword from the puzzle is the code to a code box where another puzzle is hidden. | Learning goal 9: knowing relevant infectious diseases and their transmission path. NKLM learning goals: VII.1b-02.5.23; V.01.1.1.71 |
| 50-60 | Endangerment of child welfare | -Students should notice children’s games around and flag this to the police  -on the desk they find an incomplete information leaflet on child endangerment where they must fill in the blanks | -Learning goal 12: understanding (early) signs of physical and psychological child abuse  -Learning goal 13: knowing their obligations, where to turn to and when they can break their confidentiality as doctors if a child is at risk |
| 50-60 | Medication | Spread around in the room lie many boxes of different medications and an incomplete medication plan. Students have to fill in the right medication or purpose of the drug. | Learning goal 14: identifying common medications and psychotropic drugs. NKLM learning goal: VII.3.-13.1.21 |
| 50-60 | Classes of psychotropic drugs | In one of the code boxes students find a ripped-up puzzle on different psychotropic drugs and their biological mechanism. When put together correctly on the back is a hint given about the potential perpetrator. | Learning goal 15: learning about the different biological mechanism of psychotropic drugs.  NKLM learning goal: VII.3.-13.1.21 |
| 50-60 | Addiction | -students learn about the persons drug substitution therapy and addiction in the doctors letter.  -Students find an information leaflet on addiction, how it comes about, why it is maintained and how to treat it. They have to fill in the blanks | Learning goal 16: how is an addiction diagnosed and what is the underlying biopsychological model that maintains such an addiction.  NKLM learning goal: VII.3-13.1.28; VII.3-13.5.16; VIII.4-04.3.2 |

**The order of the puzzles/task follows a vague storyline and is guided by two actors (police officers) in the room. In the psychiatry puzzles towards the end are interchangeable.**

**Copyright Disclaimer: If you want to replicate the script, please obtain written confirmation from the corresponding author**

**Sketch of the set-up of the Escape Room and surrounding facilities:**

**
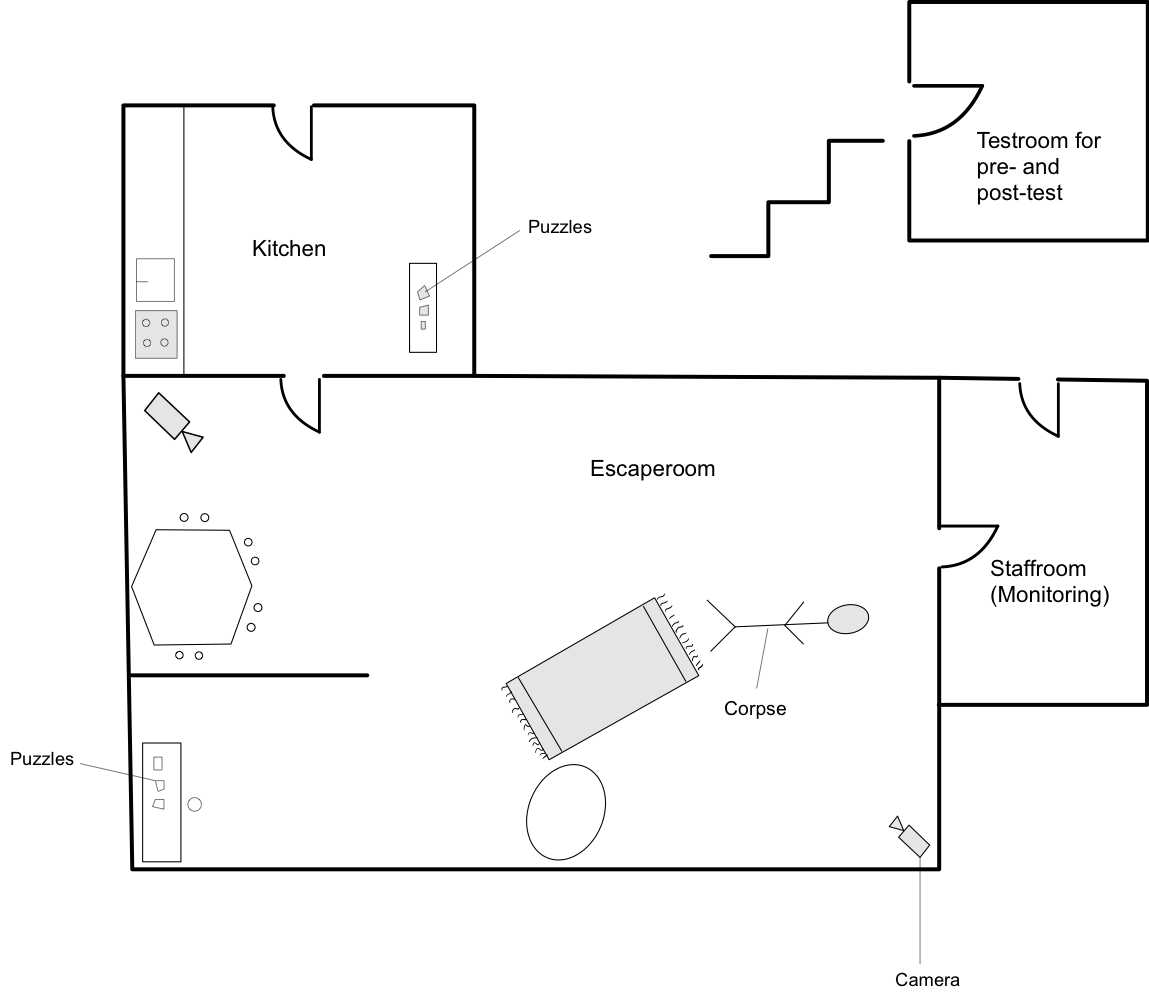
**
