# Supplementary material for: Game-Based Medical Education: Learning Effects of an Interdisciplinary and Interprofessional Escape Room
Source: Med Sci Educ. 2026 Mar 10;36(3):1593–603. doi: 10.1007/s40670-026-02661-3 (PMC13355985; doi:10.1007/s40670-026-02661-3)
Supplement: Supplementary file 2 — Appendix B [file 40670_2026_2661_MOESM2_ESM.docx]

**MitMach Tatort Story**

### Minutes 00-30: Briefing

In a separate room students receive a short introduction to the escape room and the rules. Afterwards they undertake a 20 minute pre-test. Once all students are done with the test and all open questions have been answered, the students get picked up by two co-game masters in the role of police officers. The police ask the students, who take on the role of a general practitioner, to help them out with a patient of theirs. The police explain to the students that they have been called by a worried neighbour to the house of Miss Schmidt because he has heard a loud argument an hour before. The police went to check on Miss Schmidt and found her lying unresponsive on the floor. They then right away came to the students to ask for help and to accompany them to miss Schmidt’s house.

### Minutes 30-35: Prologue

The students enter the flat of miss Schmidt, which consists of a kitchen and a living room. Upon entering the students see Miss Schmidt (a medical dummy doll) lying on the floor with the police commenting that they have found her like that. They couldn't feel a pulse and she seems pretty cold already (hinting at uncertain signs of death). The police assume that no reanimation is necessary anymore, the students themselves should then see for themselves if they can find certain signs of death and whether a reanimation really isn't necessary anymore.

### Minutes 35-60: entering the crime scene (forensic medicine)

### The students confirm the person's death by identifying certain signs of death (Rigor mortis, livor mortis etc., the doll has been accordingly modified). Once the death has been confirmed the police officers ask the students to do the post mortem examination and fill out the death certificate. The police hands the students a camera asking them to document their findings. After some time one police officer asks for the camera back and heads out to get the photos printed. The officer comes back after some time with a handful of printed magnetic pictures of photos of real death signs and wound types that have to be matched to the correct description. The police officer hands the photo-puzzle over, mentioning that the order got mixed up and whether the students can please help bring it in the right order again. The police officer also asks the students what they think about how the person died. In the death certificate the students have to choose between a natural or unnatural cause of death. Even though it becomes apparent throughout the post mortem examination that there has been an external force to the head and an unnatural death cause is likely, the police officers pressure the students to note down a natural cause of death so they have less work. Students should fill out the death certificate correctly and identify that it’s an unnatural or unknown cause of death.

### Minutes 60-75: Infectiology

The students get interrupted by an outburst of anger and fear of one of the police officers that discovers blood of the victim on his/her hand. The two officers get into a discussion, whether she now might have caught an infectious disease. Apparently one of their colleagues caught something a few weeks ago. Scared and slightly anxious the police officer asks the students what kind of diseases the victim might have had. And what the chances are that it might have been transmitted. The students then should start looking for a doctor's letter or any other information about the victim. While scanning the room they find a doctor's letter that informs them about the victim's drug addiction and other mental health issues but no previous infectious diseases. Also they find an infectiology crossword puzzle that they should fill out. Filled in correctly the crossword puzzle provides them with a codeword for a code box. The code box is hidden in the room and contains another medical quizz about psychopharmaceuticals. The police officer gets out her vaccination passport and asks the students to check if she is missing any crucial vaccinations for her job as a police officer. The students should recognize that one hepatitis B vaccination is missing, which is an important vaccination for people that get in touch with potentially infectious liquids.

### Minutes 75-85: psychiatric background

After the police officer has calmed down, they start to ask the students about any information they have found about the victim. Since they assume an unnatural death they ask about a potential course of events that has led to the death of miss Schmidt. The students then should start exploring the surroundings. In the living room children's toys can be found which lead to the question if children are involved and where they are. On the table students find two information brochures about child endangerment and drug addiction that are incomplete. The students should then fill in the missing information learning about risk factors for and legal obligations in cases of child endangerment and drug addiction. Also the students will find a codebox which contains a puzzle about psychopharmaceuticals. Throughout the room are small hints hidden about the biography of the victim and the potential perpetrator. On the working desk are various medicine boxes and an incomplete medication plan. By filling in the medication plan correctly the students receive another code to a codebox. Whenever the students get stuck or don´t find certain hidden clues the police officers give them subtle hints.

### Minutes 75-80: Finding the perpetrator and the weapon

After solving all psychiatric puzzles the students will find hints about the potential perpetrator and especially the potential weapon. The police officers ask the students whom they think it could have been and whether they found any clues. In the last code box they find a hint to use the lamp on the table. The lamp has a black light bulb and uncovers a secret note on the white board. The note reads a recipe which directs the students towards the kitchen. In the kitchen they find a cake recipe where all the ingredients but one are lying around. The flour is missing and is hidden in the cupboard. Inside the bag is a blood stained rock. Finding the rock concludes the escape room and the police walk in with the handcuffed perpetrator, in this case the husband.

### Minutes 80-125: Debriefing

After the crime has been solved all experts from the different medical disciplines (forensic medicine, infectiology and psychiatry) enter the room. Moderated by the game-master a debriefing session is led, where all medical themed puzzles are discussed and if necessary corrected. Afterwards the experts add some of their observations and give the group feedback. All open questions are addressed.
